# Effective MAPK Inhibition is critical for therapeutic responses in colorectal cancer with BRAF mutations

**DOI:** 10.1080/23723556.2015.1048405

**Published:** 2015-05-21

**Authors:** Leanne G. Ahronian, Ryan B. Corcoran

**Affiliations:** a Massachusetts General Hospital Cancer Center, Boston, MA, USA; b Department of Medicine, Harvard Medical School, Boston, MA, USA

**Keywords:** Clinical trials, colorectal cancer, BRAF, BRAF inhibitor, ERK inhibitor, MAPK, resistance

## Abstract

RAF inhibitor monotherapy is ineffective in *BRAF*-mutant colorectal cancer (CRC) but RAF inhibitor combinations have demonstrated improved efficacy, likely through superior suppression of MAPK signaling. The first identified mechanisms of acquired resistance to these combinations all promote MAPK reactivation, underscoring the MAPK pathway as a critical target in *BRAF*-mutant CRC.

Activating mutations in *BRAF* occur in approximately 10% of colorectal cancers (CRCs)^1^ and trigger constitutive activation of the mitogen-activated protein kinase (MAPK) pathway. Notably, *BRAF* mutations in CRC confer a poor prognosis,^2^ therefore improved therapies for these patients are needed.

RAF inhibitor monotherapy has been effective in *BRAF*-mutant melanoma, producing response rates of approximately 50–80%.^3^ However, the same treatment in *BRAF*-mutant CRC yielded a response rate of only 5%.^4^ This difference in sensitivity suggests that resistance in *BRAF*-mutant CRC is driven by unique signals. Defining these resistance signals may reveal opportunities to improve therapy.

Initially, it was hypothesized that resistance to RAF inhibitor monotherapy in *BRAF*-mutant CRC suggested a lower dependence on MAPK signaling, possibly through activation of a parallel signaling pathway. However, comparisons between *BRAF*-mutant melanoma and CRC cell lines revealed that RAF inhibitors led to sustained suppression of mitogen-activated protein kinase (MAPK) signaling in melanoma cells, whereas CRCs exhibited only transient suppression of the pathway.^5,6^ This finding suggested that *BRAF*-mutant CRCs may still be dependent on MAPK signaling, and that incomplete suppression by RAF inhibitors may be the reason behind their lack of efficacy in CRC. Thus, therapeutic strategies capable of enhancing MAPK suppression might have improved efficacy in *BRAF*-mutant CRC.

Studies by our group and others found that in many *BRAF*-mutant CRCs, RAF inhibitor-induced reductions in MAPK signaling lead to inactivation of negative feedback signals downstream of ERK, allowing epidermal growth factor receptor (EGFR) to reactivate MAPK through RAS and CRAF^5,7^ (Fig. 1A, B). EGFR levels are higher in BRAF-mutant CRCs than in melanomas, explaining why CRCs exhibit EGFR-mediated resistance more frequently.

**Figure 1.**
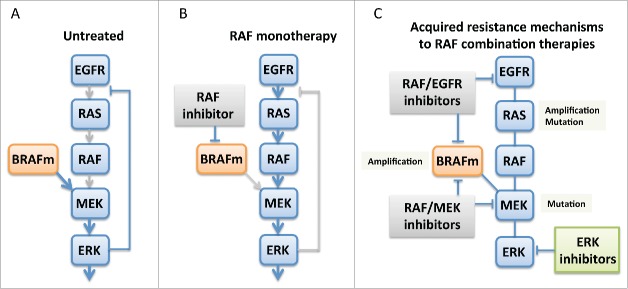
RAF inhibitors for the treatment of colorectal carcinomas bearing *BRAF* mutations. (A) Constitutively active BRAF strongly activates MAPK signaling, leading to ERK activation. Downstream of ERK, inhibitory signals reduce upstream inputs into MAPK. (B) With RAF inhibitor monotherapy, activity of mutant BRAF is reduced. This decreases the inhibitory feedback that typically occurs downstream of ERK and allows activation of RAS and wild-type RAF, leading to reactivation of MAPK signaling. (C) Mechanisms of clinical acquired resistance emerge in patients with *BRAF*-mutant colorectal cancer treated with RAF/EGFR or RAF/MEK inhibitor combinations. Despite these resistance mechanisms, ERK inhibition can suppress MAPK signaling and overcome resistance in laboratory models.

Since feedback reactivation of MAPK signaling is important for resistance to RAF inhibitors in CRC, RAF-based inhibitor combinations were tested in *BRAF*-mutant CRC laboratory models, demonstrating reduced feedback activation and improved MAPK suppression.^5-7^

Based on these data, clinical trials of RAF/MEK and RAF/EGFR inhibitor combinations were initiated in patients with *BRAF*-mutant CRC, yielding increased response rates of 12%, and 13–26% respectively.^8,9^ However, although response rates have improved, the efficacy is still lower than in melanoma. Importantly, evaluation of paired pre-treatment and on-treatment biopsies revealed that, despite inhibition of multiple pathway targets, the degree of MAPK inhibition achieved in patients with *BRAF*-mutant CRC is less than that observed in patients with melanoma treated with RAF inhibitor alone.^8,9^ These data suggest that incomplete MAPK inhibition may still limit the efficacy of these combinations.

Another strategy currently in clinical trials for *BRAF*-mutant CRC employs a combination of RAF/MEK/EGFR inhibitors.^9^ Since not all MAPK feedback occurs through EGFR, the addition of a MEK inhibitor may reduce pathway reactivation and improve MAPK suppression. Indeed, in patients receiving this therapy the degree of MAPK inhibition is comparable to that achieved in *BRAF*-mutant melanoma patients receiving RAF monotherapy.^9^ Initial response rates with the triple combination are approximately 40%, demonstrating that greater MAPK suppression can improve efficacy in *BRAF*-mutant CRC.^9^

Recently, our group identified the first mechanisms of clinical acquired resistance to RAF inhibitor combinations in patients with *BRAF*-mutant CRC. We investigated acquired resistance to RAF/EGFR or RAF/MEK combinations in 3 patients who had initially responded to these therapies. To identify new alterations that may be driving acquired resistance, a post-progression tumor biopsy was obtained and compared to a paired pre-treatment tumor biopsy by whole-exome and transcriptome sequencing. Strikingly, resistant tumors in all 3 patients had developed alterations that reactivated the MAPK pathway, once again highlighting the critical dependence of *BRAF*-mutant CRC on MAPK signaling^10^ (Fig. 1C).

The first patient progressed following an initial response to a RAF/MEK combination and was switched to a RAF/EGFR combination, on which one lesion grew rapidly throughout therapy. Comparison of the post-RAF/EGFR biopsy with prior biopsies revealed 25-fold amplification of wild-type *KRAS* in the progressing lesion that was not present in the earlier samples, implicating *KRAS* amplification as the cause of acquired resistance. Interestingly, *in vitro* modeling of acquired resistance to RAF/EGFR or RAF/MEK in *BRAF*-mutant CRC cells revealed that resistant lines had acquired activating mutations in *KRAS*, supporting the notion that KRAS activation can drive resistance to these therapies.

The second patient progressed after initially responding to a RAF/EGFR combination. A post-progression tumor biopsy was found to have high-level amplification of the *BRAF*^*V600E*^ allele, which was not present in a pre-treatment biopsy from the same lesion, as the likely resistance mechanism.

Whole-exome sequencing of post-progression biopsy from a third patient following an initial response to RAF/MEK therapy revealed the emergence of a *MEK1*^*F53L*^ mutation that was absent in the pre-treatment biopsy.

Laboratory studies confirmed that each of the alterations found in post-progression patient biopsies induced resistance to either RAF/EGFR or RAF/MEK inhibitor combinations by maintaining MAPK signaling despite therapy. This finding further emphasizes that robust suppression of MAPK signaling is critical for clinical benefit in *BRAF*-mutant CRC.

Importantly, for each identified mechanism of acquired resistance, an ERK inhibitor retained the ability to suppress MAPK signaling and could overcome the resistance. Together, these data confirm the critical dependence of *BRAF*-mutant CRCs on MAPK signaling and suggest that ERK inhibitors might become important components of future therapeutic strategies for this disease.

Overall, an understanding of the resistance mechanisms operant in *BRAF*-mutant CRC has led to improved response rates in clinical trials over the past few years. Although additional pathways may play a role in resistance of *BRAF*-mutant CRC, the data suggest that robust inhibition of MAPK signaling is of primary importance. Targeted combination therapy designed to more effectively block feedback reactivation of MAPK signaling, perhaps through the incorporation of ERK inhibitors, has the potential for improved clinical benefit in patients with this aggressive CRC subtype.
